# An Investigation into the Perceptions of Veterinarians towards Perioperative Pain Management in Calves

**DOI:** 10.3390/ani11071882

**Published:** 2021-06-24

**Authors:** Ria van Dyke, Melanie Connor, Amy Miele

**Affiliations:** Division of Animal Behaviour and Welfare, The Royal (Dick) School of Veterinary Studies, University of Edinburgh, Roslin EH25 9RG, UK; ria.vandyke@gmail.com (R.v.D.); m.connor@irri.org (M.C.)

**Keywords:** animal welfare, calves, veterinarians, pain, animal husbandry, pain management, perceptions

## Abstract

**Simple Summary:**

Despite developments in animal welfare science regarding perioperative pain management in calves (*Bos taurus*), there are concerns that current knowledge has not been adopted in practice. Given that the perceptions of veterinarians have implications for how the welfare needs of calves are assessed and managed in practice, this study sought to quantify veterinary perceptions towards perioperative pain management in calves, including barriers to its use and whether demographic differences may influence those perceptions. A nationwide survey was electronically distributed to veterinarians registered with the Veterinary Council of New Zealand. Veterinarians largely associated multimodal pain management with the greatest reduction in perioperative pain. Most veterinarians also perceived that postprocedural pain persists beyond 24 h for disbudding and castration and did not support the use of differential treatment based on developmental age. Despite this, certain barriers were identified for their potential to inhibit the use of pain management on-farm. While demographic differences were found to influence veterinary perceptions towards perioperative pain management, the findings revealed considerable support among veterinarians for improving pain mitigation in calves. Given the opportunity, veterinarians in New Zealand would likely support strengthening the minimum provisions afforded to calves in practice and policy.

**Abstract:**

While veterinarians are instrumental to the welfare of calves (*Bos taurus*), limited knowledge exists concerning veterinary perceptions towards perioperative pain management in calves. As a part of a larger, nationwide study investigating the perceptions of veterinarians towards calf welfare, the current work sought to quantify veterinary perceptions towards perioperative pain management, including barriers to its use, and investigate demographic influences affecting those perceptions. An electronic mixed-methods survey was completed by 104 veterinarians registered with the Veterinary Council of New Zealand. The current work revealed that most veterinarians considered a multimodal approach as the most effective method for ameliorating perioperative pain in calves, rejected the practice of differential treatment based on developmental age, and perceived that postprocedural pain persists beyond 24 h for the majority of procedures included in the survey. Despite this, veterinarians identified certain barriers that may inhibit the provision of pain mitigation on-farm, including costs, inadequate recognition of pain, and ingrained farming practices. Certain demographic effects were found to influence perceptions towards perioperative pain management, including gender, the number of years since graduation, and species emphasis. Nevertheless, the current work demonstrated considerable support among veterinarians to improve pain management protocols during routine husbandry procedures. The asymmetries that exist between the current minimum provisions of perioperative pain management and veterinary perspectives suggest that substantive improvements are necessary in order to reconcile New Zealand’s existing regulatory regime with developments in scientific knowledge.

## 1. Introduction

Unmitigated pain is associated with suffering and distress, and thus represents a critical welfare concern within farm animal practice [1,2,3]. While freedom from pain is considered a requisite for animal welfare [4,5,6], calves (*Bos taurus*) are often subjected to painful husbandry procedures without the provision of pain relief [7,8,9,10]. Young calves are particularly vulnerable to welfare compromise [11,12,13]. Given that routine husbandry procedures are often performed on calves at an early age without a developed adaptive immune system [14], painful stressors may suppress immune function and increase susceptibility to disease in immunologically naïve neonates [14,15,16]. Evidence suggests that pain mitigation may attenuate suppressed leukocyte function during such routine procedures in calves [16]. There is concern that unmitigated pain may lead to incapacitating pathophysiological effects that not only compromise welfare, but also increase the risk of morbidity and mortality [13,17]. Furthermore, there is evidence that noxious experiences during neonatal development may have systemic effects on nociceptive processing, resulting in hypersensitivity to pain later in life [18,19,20].

Invasive husbandry procedures are routinely performed on very young animals without pain relief on the basis of a misconception that younger animals experience less pain [21]. While developmental differences in the experience of pain have been studied explicitly with calves using behavioural and physiological indicators [18,22,23], such age-based comparisons are limited due to a fundamental problem with interpretation. Developmental changes in young animals may include changes in the sensitivity of certain indices used to assess pain, such as cortisol [4,24,25]. For this reason, different responses to the same pain index at different developmental ages does not definitively demonstrate changes in the severity of pain experienced [24]. While local anaesthesia has been found effective in managing the acute nociceptive pain during painful husbandry procedures [26,27,28], postprocedural pain arises due to the inflammatory processes initiated by tissue damage [27,29]. Given the complexity of the mechanisms involved in nociception, a multimodal approach that involves the administration of pharmaceutical agents before, during, and/or after a procedure has been described as best practice in mitigating perioperative pain [21,27,29,30,31,32,33,34]. Despite this, multimodal pain management is exceedingly rare in farm animal practice [21,29,35,36].

Given that an association has been found between veterinary perceptions towards pain and the use of analgesic agents in practice [37,38], the perceptions of veterinarians towards the welfare needs of calves have direct implications for how those needs are assessed and managed in practice [6,35]. Despite extensive research on the importance of multimodal protocols in the management of pain in companion animals [21,28,29,39,40,41], there are comparatively few studies on veterinary perspectives towards the management of perioperative pain in bovine species. Of the research available, most studies are focused on attitudes towards the acute pain phase associated with certain husbandry procedures [42,43] and painful conditions [38,43,44] in dairy cattle. In particular, very few studies have investigated veterinary perspectives towards a multimodal approach to pain management in calves [35,36]. Further, there are no published studies that have explored whether veterinarians support differential treatment based on developmental age, and this warrants further investigation.

The objective of this study was to investigate the perceptions of veterinarians towards perioperative pain management in calves. More specifically, this study sought: (i) to determine how different pain mitigation protocols are perceived across certain husbandry procedures; (ii) to examine the perceptions of veterinarians towards postprocedural pain in calves; (iii) to explore whether veterinarians support differential treatment based on the developmental age of a calf; (iv) to identify areas thought by veterinarians to serve as barriers to the provision of pain management on-farm; and (v) to determine whether certain demographic factors influence those perceptions. Meeting these research objectives will enable the findings to be used by researchers and educators to identify areas where veterinary estimation of pain in calves does not align with scientific knowledge, and by legislators as a measure of expert consensus on perioperative pain associated with routine husbandry procedures in calves.

## 2. Materials and Methods

Prior to commencement, ethical approval was obtained from the University of Edinburgh Human Ethical Review Committee (HERC_269-18).

### 2.1. Survey Development

Facilitated by the existing literature, a survey was developed through open dialogue with veterinarians, academics, and veterinary students. Initial pilot interviews were implemented with a small sample of veterinary students to test the survey for applicability and comprehensibility. The survey was then electronically distributed (Jisc Online Surveys) to a second sample of students for the purpose of pilot testing. In order to determine whether certain demographic effects influence perceptions towards perioperative pain management, the final version of the survey collected the following quantifiable characteristics: gender, birth year, graduation year, and species emphasis.

Respondents were asked to provide a pain score for different routine husbandry scenarios presented as single-item rating scales. While pain scales are a subjective measure, they offer an important contribution to pain evaluation because such measures enable the majority opinion of multiple informed assessors to be quantified [38] and provide valuable insights into where pain estimation may not align with current scientific knowledge [43]. The scenarios varied by husbandry procedure, the developmental age of the calf, and the type of pain relief administered. The husbandry procedures included: castration with rubber rings, castration with high tension bands, disbudding, and supernumerary teat removal. These procedures were selected due to their identification in the literature as painful [2,45,46,47]. The scenarios were based on regulatory proposals developed by the Ministry for Primary Industries (MPI) [48] or considered current practice at the time of survey development [49] (Table 1). To investigate perceptions towards multimodal pain management, respondents were asked to score pain depending on the presence or absence of local anaesthesia and/or postoperative analgesia. A 10-point rating scale was used to minimise the potential for central tendency bias [50]. Responses were assigned an incremental numerical value (1 = no pain at all, 10 = most severe pain).

Respondents were then asked to indicate the likely duration of postprocedural pain in calves across these husbandry procedures through the use of multiple-choice questions. This measure was adapted from Hambleton and Gibson’s study [42], which investigated the opinions of veterinarians towards post-disbudding analgesia in calves, and modified to include the procedures in the scenario-based questions.

For the purpose of investigating whether veterinarians support differential treatment based on the developmental age of a calf, respondents were asked to provide the age beyond which it is necessary to provide pre-emptive local anaesthesia and/or postoperative analgesia for certain procedures through the use of multiple-choice questions.

In order to explore veterinary perceptions towards barriers to the provision of pain management on-farm, respondents were asked to identify which factors may serve as barriers to the use of pain relief among farmers. A 6-point Likert-type scale was developed to reduce the potential for neutral responding (1 = strongly disagree, 6 = strongly agree). Potential barriers were selected based on their identification in the literature (Table 2). Respondents were given the opportunity to elaborate further if desired.

### 2.2. Sampling

The Veterinary Council of New Zealand (VCNZ) granted permission to recruit practising veterinarians. A list of 604 veterinarians was retrieved and compiled into a database (Microsoft Excel 2016). Inclusion in the database was based on veterinarians who displayed a direct e-mail address and were listed as currently operating in clinical practice in New Zealand. Given that the present study was developed in the wake of intense scrutiny towards calf welfare in New Zealand [11,67], along with subsequent legislative transformation, anonymous self-administration was enabled to reduce the perception of personal risk and enhance self-disclosure [68,69]. A cover letter introducing the nature of the research, along with a link to the survey (Jisc Online Surveys), was administered to all veterinarians included in the final database. This was followed two weeks later with a courtesy message thanking those who had responded and appealing to others to respond. This approach follows the general recommendations for survey protocol advocated by Dillman [70].

### 2.3. Data Analysis

Data was exported into the Statistical Package for the Social Sciences (SPSS Version 24). Main effects and interaction effects were considered significant at *p* < 0.05. Sample demographics were reported as descriptive statistics. To explore gender differences for continuous variables, an independent samples t-test was utilised, with effect size reported as Cohen’s *d*. For analysis of perceptions towards different pain management strategies, a general linear mixed model (GLMM) was fitted with the restricted maximum likelihood (REML) method to explore the factors affecting perceived pain ratings. The model was fitted with demographic factors, along with pain management strategy, as fixed effects and participant unique identifiers as a random effect. Pairwise interaction effects were investigated between all factors when preparing the model. Through multiple iterations, the model was simplified until only main effects and significant interactions remained. The significance of fixed effects was determined by type III Wald tests for main effects and type I for interaction effects. In order to explore the influence of demographic effects on perceptions towards postprocedural pain durations, ordinal logistic regression (OLR) was utilised. Odds ratios (ORs) were calculated as a measure of estimated effect size. The assumption of proportional odds was evaluated through the test of parallel lines, and multicollinearity was assessed using variance inflation factor (VIF). Perceptions regarding differential treatment were reported as descriptive statistics. Binary logistic regression (BLR) was then used to explore the influence of demographic effects on those responses. Response categories were consolidated to ensure the assumption of independence of irrelevant alternatives was met. A Spearman’s rank order correlation was used to explore whether there was an association between perceptions towards postprocedural pain durations and differential treatment. Likert-type responses regarding barriers to the provision of pain management on-farm were reported as descriptive statistics. Mann–Whitney U tests were used for two sample comparisons (e.g., gender) and Kruskal–Wallis H tests for multilevel samples (e.g., years since graduation, species emphasis). Significance levels were subject to Bonferroni correction to reduce the impact of Type I errors. Homogeneity of variance was analysed using Levene’s test. Multicollinearity was assessed through VIF. Respondents were also given the opportunity to elaborate further and responses which were most widely shared among veterinarians were reported.

## 3. Results

Of the 106 surveys returned, one submission with all missing entries, along with another submission with only demographic data provided, were excluded from the study. In total, 104 veterinarians were included in the final dataset.

### 3.1. Demographic Data

**Figure 1:**
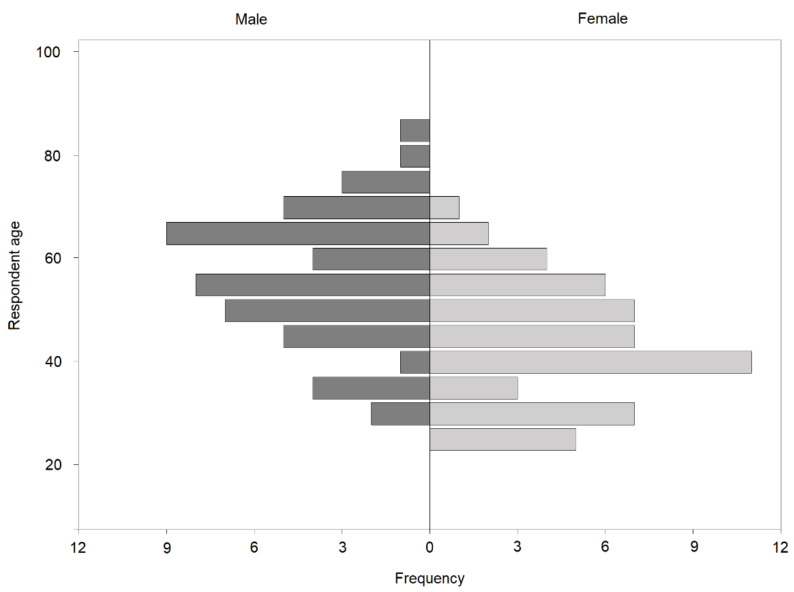
Gender and age distribution of veterinary respondents.

A breakdown of the sample demographics is included in Table 3. As might be expected there was a strong correlation between respondent age and years since graduation (r = 0.96; *p* < 0.001). Independent samples t-tests demonstrated that female veterinarians graduated more recently than male veterinarians (*p* < 0.001) and were also significantly younger (*p* < 0.001; Figure 1). Chi-squared tests indicated that there were no significant differences between the number of male and female respondents specialised in a certain species (χ^2^ (2) = 4.53, *p* = 0.104).

### 3.2. Perceptions towards Perioperative Pain Management

The GLMM model was fitted with demographic factors, pain management strategies, and pairwise interactions as fixed effects. The model determined that the effect of pain management was highly significant across all procedures (all *p* < 0.001), with multimodal pain mitigation associated with the greatest reduction of perceived pain (Table 4).

Across all pain management strategies, female veterinarians had significantly higher perceived pain scores than male veterinarians for supernumerary teat removal (*F*_1,452_ = 6.92; *p* = 0.009) and disbudding (*F*_1,377_ = 8.80; *p* = 0.003). There was also a significant effect of the number of years since graduation on perceived pain scores for disbudding (*F*_1,388_ = 17.07; *p* < 0.001), which indicates that more recently graduated respondents perceived more pain. Across all procedures, an interaction effect was found between gender and years since graduation (Table 5). Male veterinarians had lower pain scores as the number of years since graduation increased (all *p* < 0.05; Table 5). No significant effect was found between years since graduation and females across all procedures (*p* > 0.05), which suggests that pain ratings were consistent among females, irrespective of the number of years since graduation. Significant differences in perceived pain scores for castration with rubber rings (*F*_2,860_ = 5.36; *p* = 0.005) and supernumerary teat removal (*F*_2,452_ = 23.62; *p* < 0.001) were found between veterinarians working in the three types of veterinary practice, with the largest differences found between veterinarians working in companion animal practice and veterinarians working in large animal practice (Table 6).

### 3.3. Perceptions towards Postprocedural Pain

Veterinarians were asked for their opinion on the likely duration of postprocedural pain following four painful husbandry procedures (Table 7). Most veterinarians perceived that postprocedural pain persists beyond 24 h for disbudding (74.5%; *n* = 76; $\bar{X}$ = 5.01; $\sigma_{\bar{x}}$ = 0.12), castration with rubber rings (65.5%; *n* = 66; $\bar{X}$ = 4.51; $\sigma_{\bar{x}}$ = 0.15), and castration with high tension bands (63.3%; *n* = 57; $\bar{X}$ = 4.56; $\sigma_{\bar{x}}$ = 0.15). There was less agreement between responses for supernumerary teat removal, with most veterinarians perceiving pain durations of less than 24 h (65.6%; *n* = 63; $\bar{X}$ = 3.72; $\sigma_{\bar{x}}$ = 0.15).

An OLR model, fitted with demographic factors, demonstrated that across all procedures, an increase in years since graduation was associated with lower perceived postprocedural pain durations (all *p* < 0.05; Table 8). Species emphasis also had a significant effect on veterinary perceptions towards postprocedural pain. With the exception of disbudding, veterinarians working in large animal practice were less likely to perceive greater postprocedural pain durations than veterinarians working in companion animal practice (all *p* < 0.05; Table 9). Veterinarians working in large animal practice were also less likely to perceive greater postprocedural pain durations for supernumerary teat removal than veterinarians working in mixed animal practice (*p* = 0.008; Table 9). The effect of gender on perceptions of postprocedural pain was not found to be significant (*p* > 0.05).

### 3.4. Perceptions towards Differential Treatment

Veterinarians were asked for their opinion on the age beyond which it is necessary to provide pre-emptive local anaesthesia and/or postoperative analgesia in calves across certain painful procedures. With regard to the procedures that do not legally require local anaesthesia, the majority of veterinarians perceived that local anaesthesia should be provided for both castration with rubber rings (59.2%; *n* = 61) and supernumerary teat removal (57.1%; *n* = 56), irrespective of developmental age. Further, the greatest proportion of veterinarians supported the use of postoperative analgesia at any age for disbudding (68.3%; *n* = 69), castration with high-tension bands (64.4%; *n* = 57), castration with rubber-rings (55.3%; *n* = 57), and supernumerary teat removal (44.3%; *n* = 43) (Table 10). Spearman’s rank order correlation demonstrated that across all procedures, a strong correlation was found between veterinary perceptions towards postprocedural pain and the age beyond which calves should be provided with postoperative analgesia (all *p* < 0.001). This finding demonstrates that veterinarians that perceived greater postprocedural pain durations were likely to indicate stronger support for the use of postoperative analgesia at any developmental age.

A BLR model, fitted with demographic factors, determined that veterinarians working in companion animal practice were 3.64 times more likely to perceive the provision of local anaesthesia as necessary for castration with rubber rings and 7.35 times more likely for supernumerary teat removal than veterinarians working in large animal practice (95% CI (1.08, 12.20); *p* = 0.037 and 95% CI (2.02, 26.03); *p* = 0.002, respectively). Further, veterinarians working in companion animal practice were 7.19 times more likely to perceive the provision of postoperative analgesia necessary for supernumerary teat removal at any developmental age than veterinarians working in large animal practice (95% CI (2.14, 23.81); *p* = 0.001). A per year increase in years since graduation was associated with a 0.93 times reduction in odds that veterinarians would perceive postoperative analgesic as necessary at any age following disbudding (95% CI (0.89, 0.97); *p* < 0.001). The effect of gender on perceptions towards differential treatment was not found to be significant (all *p* > 0.05).

### 3.5. Perceived Barriers to the Provision of Pain Management

**Figure 2:**
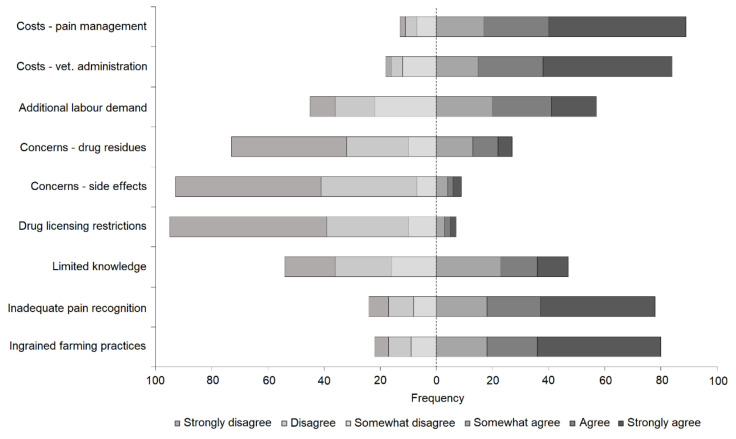
The number of veterinarians that perceived certain factors as barriers to the provision of pain management on-farm, where the dashed line represents the point between disagreement and agreement based on responses to a 6-point Likert-type scale.

Perceived barriers to the provision of pain management on-farm with the greatest level of agreement among veterinarians included the costs associated with pain management, the costs associated with veterinary administration, inadequate recognition of pain, and ingrained practices (Figure 2). The barriers perceived as least likely to influence the provision of pain relief on-farm included concerns for the impact of drug residues on production quality and concerns that drugs may have side effects (Figure 2). Mann–Whitney U tests indicated that female veterinarians were significantly more likely than male veterinarians to perceive ingrained farm practices as a barrier to the provision of pain management (*p* = 0.033). Kruskal–Wallis H tests, followed by Mann–Whitney U tests for post hoc pairwise comparisons with Bonferroni correction (0.05/10 = 0.005), showed that inadequate recognition of pain was perceived to be a greater barrier to the provision of pain relief on-farm by veterinarians who had graduated within the past 20 years compared to more senior veterinarians (0–10 years: *p* = 0.002; 11–20 years: *p* < 0.001). Further, veterinarians that had graduated within the past 10 years perceived ingrained farming practices as a greater barrier to the provision of pain relief on-farm than their more senior peers (*p* = 0.003Post hoc pairwise comparisons with Bonferroni correction (0.05/3 = 0.017) undertaken with the three practice types demonstrated that veterinarians working in small animal practice were significantly more likely to perceive that concerns regarding drug residues on production quality may present a barrier to the provision of pain relief on-farm than veterinarians working in large animal practice (*p* = 0.003).

Respondents were given the opportunity to elaborate on additional barriers to the provision of pain management that were not included in the scale. Of the 16 qualitative responses, the most representative considerations included concerns for how restricted veterinary medicine (RVM) may limit the use of pain management on-farm, with lay contractors requiring “a system/authorisation to access and use RVM”. Further concerns included a “lack of understanding of the benefits of pain relief beyond just welfare”, a “lack of empathy”, and “disorganisation” on-farm. Respondents were also concerned with the normalisation of procedures without pain mitigation due to the accessibility of lay contractors “with apparent expertise advertising services without pain relief, which legitimises the practice”.

## 4. Discussion

This paper is part of a larger, nationwide study exploring veterinary perceptions towards calf welfare in New Zealand [67]. The aim of this study was to investigate current thinking among veterinarians towards perioperative pain in calves.

### 4.1. Perceptions towards Perioperative Pain Management

Veterinarians in the current study associated multimodal pain management with a significant reduction of pain in calves when compared with no pain relief or local anaesthesia alone, which is consistent with the literature [27,33,71,72]. In line with scientific knowledge, the New Zealand Veterinary Association has identified that husbandry procedures that involve tissue damage are painful, affirming that analgesia must be included in the planning for all surgical procedures and continued for an appropriate duration following the procedure [73]. Although the benefits of multimodal protocols are well-established [21,27,29,30,31,32], multimodal pain management in calves is seldom used in practice [21,29,35,36], which raises concerns regarding barriers to its’ use.

In the current work, female veterinarians scored pain significantly higher than male veterinarians for supernumerary teat removal and disbudding, a finding that has been frequently reported [36,38,42,43,62,74,75]. While the role of empathy is outside the scope of the current work, previous studies have found that empathy may underpin perceptions of animal pain [63,76], with a greater capacity among females to engage in affective resonance with animals [77,78]. In line with previous studies [38,42,43,65,74,75,79], more recent veterinary graduates scored pain higher than their more senior colleagues for disbudding. Moreover, more recently graduated males perceived greater pain than their male colleagues. These findings may indicate that more recent veterinary graduates have greater access to developments in the scientific literature or that a greater emphasis has been placed on perioperative pain in the veterinary curricula in recent years. Veterinarians working in companion and mixed animal practice scored pain higher than veterinarians working in large animal practice across half of the procedures in question. While veterinarians are trained to consider animal health at the individual level, veterinarians in large animal practice are often tasked with managing animal health on a collective level [80]. This shift towards collective care places a greater emphasis on the utility or instrumental value of animals rather than the affective state of individual animals [81,82], which has important implications for the level of pain management afforded to calves in practice [35,37,38].

### 4.2. Perceptions towards Postprocedural Pain

The majority of veterinarians in the current work perceived that postprocedural pain persists beyond 24 h for most painful husbandry procedures in question, which is supported by the scientific literature [2,22,39,40,83,84,85]. However, where pain management is required for painful husbandry procedures, regulations often focus on the experience of acute pain, with little consideration for pain that may persist beyond the perioperative period. Very few studies have focused on veterinary perceptions towards postoperative analgesic use and the extent to which it is adopted in practice. Although earlier research has indicated that postoperative analgesic use is limited [10,38,64], there is scope for researchers to investigate how these perspectives and practices may have shifted over time. The present study explored potential barriers to the management of pain on-farm more generally, and this is discussed later (see Section 4.4).

Consistent with previous studies [38,42,43,54], more recent veterinary graduates perceived longer postprocedural pain durations than their more senior colleagues. As previously mentioned, it is important to recognise the influence of animal welfare science on the veterinary curricula over time [86,87], which reinforces the need for continuing professional development to capture emerging scientific knowledge. Moreover, veterinarians working in large animal practice perceived significantly lower postprocedural pain durations than veterinarians working in companion animal practice, with the exception of disbudding. This finding indicates that the perceptions of veterinarians towards postoperative pain may be influenced by their work type. In line with these findings, previous studies have reported that students aspiring to work in specialisations outside of large animal practice rated higher pain in cattle [75] and shared greater concern towards farm animal welfare [81] than those that elected to work in large animal practice. Given that perceptions towards postprocedural pain will have implications for the use of postoperative pain relief in practice [37,38], there is scope for further investigation into these trends within the profession.

### 4.3. Perceptions towards Differential Treatment

Veterinarians in the present study largely perceived that all calves have the capacity to suffer from unmitigated pain and supported a multimodal approach to ameliorating perioperative pain in calves, irrespective of developmental age. This finding reveals that most veterinarians rejected the ontogeny of sentience as a basis for differential treatment in calves and highlights the discord between current regulatory standards and veterinary perspectives. However, differential treatment based on developmental age is common practice. For instance, supernumerary teat removal can be performed on young calves under 10 weeks of age without any pain relief, despite teat removal being considered a significant surgical procedure [51]. While scientific understanding of pain in cattle has developed in recent years to recognise pain and distress in young calves following painful husbandry procedures [34,71,88,89], the use of pain relief has been reported as less frequent in younger calves [90,91]. This may be due to the misconceptions that exist in the use of pain management in young farm animals, including the belief that younger animals experience less pain [60]. It is also possible that pain relief may be used less frequently in younger calves due to the ease of handling younger animals [64,66] and the fewer regulatory welfare provisions afforded to younger calves. Despite this, across all procedures, greater perceived pain durations were strongly correlated with increased support for the use of postoperative analgesia at any developmental age. As with previous studies [38,54], this indicates that an increased ability to recognise a procedure as having the potential to cause prolonged pain is likely to result in increased administration of analgesic agents to mitigate postoperative pain. Indeed, respondents who did not support the use of postoperative analgesia assigned significantly lower scores for postprocedural pain for all procedures in question, suggesting that a key motivator for the use of analgesic agents is the veterinarian’s own perception of the pain that the animal is suffering [38].

While most veterinarians did not support differential treatment based on developmental age, certain demographic effects were found to influence those perceptions. Senior veterinarians were less likely to perceive the use of postoperative analgesia as necessary at any age following disbudding. This finding echoes Hambleton and Gibson’s [42] report that stronger support was found among more recent veterinary graduates for the compulsory use of analgesic agents to reduce post-disbudding pain in calves. Further, veterinarians working in large animal practice were less likely to perceive local anaesthesia as necessary at any developmental age for castration with rubber rings and supernumerary teat removal, and less likely to perceive postoperative analgesia to be necessary following supernumerary teat removal than veterinarians working in companion animal practice.

### 4.4. Perceived Barriers to the Provision of Pain Management

To facilitate the development of strategies targeted at improving the uptake of practices that alleviate perioperative pain, it is important to consider the barriers that may impact upon such efforts [6]. A range of barriers to the provision of pain relief on-farm were identified, including the additional costs associated with pain mitigation, which is reinforced in the literature [60,64,90,91,92,93,94]. This highlights the complex, often competing, interests that veterinarians must navigate as they balance their professional obligations to safeguard the economic interests of their clients and uphold their moral duty to intervene on behalf of the animal [80,95,96]. Respondents also expressed concerns that lay contractors may offer services without the provision of pain relief, which not only normalises the practice, but may also intensify tensions regarding costs [97]. Previous studies have found that if it is perceived that their clients are concerned with costs, veterinarians are less likely to address options for pain management [66]. However, farmers may be receptive to changing current practices if they understand that pain management is associated with improved outcomes for animal health and welfare [98]. These outcomes include significant pain alleviation [21,39,99,100], reduced pain-related behaviour [2,33,101], and increased feed intake [39,102,103,104]. In addition to the costs associated with pain mitigation and its administration, veterinarians perceived that inadequate recognition of pain and ingrained practices are barriers to the provision of pain relief on-farm. While knowledge on pain recognition in cattle has developed considerably in recent years [105,106,107], there are concerns that developments in animal welfare science may not be accessible to farmers [108], therefore limiting the scope for scientific knowledge to challenge existing practices. Given that farmers perceive veterinarians as educators and advisors on matters of animal welfare [96,109,110], veterinary–client communication plays an important role in the transfer of knowledge [96]. The ability for veterinarians to effectively translate knowledge on pain recognition, assessment, and management into on-farm application is therefore a pivotal issue worthy of greater support [98].

### 4.5. Limitations

The VCNZ relies on individual members to ensure that their details are current in their database. However, some of the electronic invitations to participate in the survey were undeliverable and returned. For this reason, the survey did not reach all members of the intended sample and a response rate cannot be accurately quantified. Despite this, the demographic data indicates a diverse range of respondents across gender, age, graduation year, and species emphasis. Furthermore, the age and gender distribution of the respondents in the current work is representative of the VCNZ Workforce Report [111].

### 4.6. Implications

Despite recognition that animals are capable of suffering from unmitigated pain, legislative protection of farm animals is limited [6,105,112,113,114,115,116,117,118]. In the current work, veterinarians associated multimodal pain relief with the greatest reduction of pain across all husbandry procedures in question. These perceptions align with current scientific knowledge [21,27,29,30,31,32,33,34] and are views shared by veterinary authorities globally [119,120,121]. However, multimodal protocols are not a legal requirement in farm animals and remain significantly underused in practice [21,29,35,36,42].

Although veterinarians in the present study rejected the use of differential treatment based on developmental age, the level of welfare protection afforded to young farm animals is inconsistent, representing a global welfare concern [34,105]. For instance, in New Zealand, rubber ring castration can be performed without any pain relief in calves up to 6 months of age [51], representing an example of policy that has not progressed with developments in animal welfare science. In contrast, rubber ring castration without an anaesthetic is limited to within the first week of life in the United Kingdom [122]. Legislation that requires the provision of pain relief after a specified developmental age underlies the assumption that calves in later stages of development are capable of suffering, and therefore require legal protection [123]. This is particularly problematic because it establishes an ontogeny of sentience that is not supported by scientific evidence.

Despite recent regulatory amendments introduced to address areas of highest risk to calf welfare in New Zealand, the resulting regulations have been criticised for favouring industry voices, incentivised to support standards that would otherwise fall below the general provisions of the Act [117,118,124]. The asymmetries that exist between current regulations and veterinary perspectives suggest that substantive changes are needed to improve New Zealand’s animal welfare regime in line with current scientific knowledge. Given that veterinarians are considered an authority on matters of animal welfare [110,125], veterinary perceptions should be used to inform the development of standards of practice [37]. The current work reinforces the importance of the veterinary voice being heard on policy decisions for which it is eminently qualified to comment [126].

## 5. Conclusions

Despite developments in animal welfare science, which have led to a greater understanding of perioperative pain in calves, substantive legislative reforms are necessary in order to reconcile New Zealand’s existing regulatory regime with veterinary perspectives. While certain demographic effects influenced perceptions towards perioperative pain management, the current work revealed considerable support for strengthening the level of welfare protection afforded to calves in New Zealand, found in veterinarians’ shared affinity for improving pain management protocols. Most veterinarians considered a multimodal approach as the most effective method for ameliorating perioperative pain, perceived that postprocedural pain persists beyond 24 h for castration and disbudding, and rejected differential treatment based on developmental age. Despite strong veterinary support for improving pain management protocols, veterinarians identified a number of barriers to pain mitigation on-farm, including costs, inadequate pain recognition, and ingrained farming practices. The knowledge gained from this research highlights the importance of the veterinary voice being heard on matters of animal welfare. Given the opportunity, veterinarians in New Zealand would likely support regulatory reform to strengthen the legal welfare protection afforded to calves in practice and policy.

## Figures and Tables

**Table 1:** Proposed welfare regulations or standards of practice in New Zealand regarding the provision of pain management for certain husbandry procedures at the time of survey development.

Procedure	Regulation or Standard
Castration with rubber rings	Castration with a rubber ring can be used under 6 months of age without pain relief. Over 6 months, local anaesthetic must be provided ^a^ (effective 1 October 2018).
Castration with high tension bands	Castration with a high-tension band must be performed with the provision of local anaesthetic at any age ^a^ (effective 1 October 2018).
Disbudding	Disbudding must be performed with the provision of local anaesthetic at any age ^a^ (effective 1 October 2019).
Supernumerary teat removal	Supernumerary teat removal can be performed under 6–10 weeks of age without pain relief. Over 6–10 weeks, pain relief is recommended ^b^ (pain relief required over 10 weeks of age, effective 27 July 2020).

^a^ Proposed calf welfare regulations [48] and ^b^ standards of practice at the time of survey development in 2018 [49]. Current calf welfare regulations as of 2021 [51] are included in parenthesis.

**Table 2:** Likert-type scale items adapted from the existing literature concerning potential barriers to the provision of pain management.

Scale Item	Adapted from
Costs of pain management	[42,44,52,53,54]
Costs of veterinary treatment	[52,55,56]
Labour demand associated with administration	[42,52,57]
Concern that drugs may affect production quality	[35,58,59,60]
Concern that drugs may have side effects	[42,44,54,60,61,62]
Drug licensing restrictions	[42,52,63]
Limited knowledge on the available options	[46,52,64,65,66]
Inadequate recognition of pain	[35,38,52,56]
Ingrained farming practices	[42,52,65]

**Table 3:** An overview of the sample demographics.

Demographic Variable	*n*	%
Years since graduation (*N* = 103; $\bar{X}$ = 23.75; σ = 14.23)		
0–10	23	22.2
11–20	21	20.5
21–30	29	28.0
31–40	15	14.7
41 and over	15	14.7
Gender (*N* = 102)		
Male	49	48.0
Female	53	52.0
Age (*N* = 103; $\bar{X}$ = 48.75; σ = 13.92)		
24–30	12	11.6
31–40	21	20.2
41–50	23	22.2
51–60	25	24.1
61 and over	25	21.4
Species emphasis (*N* = 100)		
Large animal practice	41	41.0
Mixed animal practice	37	37.0
Companion animal practice	22	22.0

**Table 4:** Estimated marginal means (EMM) and standard error of the means (SEM) for the perceived pain scores ^a^ of veterinarians across certain husbandry procedures by pain management protocol: none, local anaesthetic only (LA), or multimodal (MM) ^b^, with demographic variables modelled as fixed effects ^c^.

	Pain Management Protocol EMM (SEM)				
Procedure	None	LA	MM	*F*	*p*
Castration–rubber rings (*N* = 103)	7.86 (0.11)	5.12 (0.11)	3.25 (0.11)	*F*_2,861_ = 466.96	<0.001
Castration–high tension bands (*N* = 90)	-	5.75 (0.18)	3.88 (0.18)	*F*_1,331_ = 57.70	<0.001
Supernumerary teat removal (*N* = 97)	6.27 (0.20)	4.00 (0.13)	2.42 (0.13)	*F*_2,452_ = 119.87	<0.001
Disbudding (*N* = 102)	-	5.61 (0.14)	3.30 (0.14)	*F*_1,377_ = 136.08	<0.001

Significance: *p* < 0.05. SEM: standard error of the mean; *F*: (*F*(df_1_, df_2_) = *F*-value); df: degrees of freedom. ^a^ Perceived pain scores were measured on a 10-point scale (1 = no pain at all; 10 = most severe pain). ^b^ For each procedure, the estimated effect of pain management protocol was compared between the minimum legislative requirement (none or LA) and MM (local anaesthesia and postoperative analgesia). ^c^ Demographic fixed effects: gender, the number of years since graduation, and species emphasis. In order to ensure the survey did not contravene the minimum requirements under the Animal Welfare Act 1999, certain response options were not provided, and these are indicated by a dashed line.

**Table 5:** The interaction effect between gender and years since graduation on the perceived pain scores ^a^ of veterinarians across certain husbandry procedures.

		Gender × Years Since Graduation	
Procedure	Gender	*T*	*p*
Castration—rubber rings (*N* = 103)	Male	*t*(860) = −3.45	0.001
	Female	*t*(860) = 0.84	0.404
Castration—high tension bands (*N* = 90)	Male	*t*(330) = −4.45	<0.001
	Female	*t*(330) = 0.51	0.614
Supernumerary teat removal (*N* = 97)	Male	*t*(451) = −1.42	0.157
	Female	*t*(451) = 1.152	0.131
Disbudding (*N* = 102)	Male	*t*(376) = −4.02	<0.001
	Female	*t*(376) = −0.55	0.581

Significance: *p* < 0.05; Direction of effect: negative *t* values indicate that senior veterinarians perceived lower pain scores than their more recently graduated peers. ^a^ Perceived pain scores were measured on a 10-point scale (1 = no pain at all; 10 = most severe pain).

**Table 6:** Estimated marginal means (EMM) and standard errors of the mean (SEM) for the perceived pain scores ^a^ of veterinarians across certain husbandry procedures by species emphasis.

	Species Emphasis EMM (SEM)						
Procedure	COM	MIX	LGE		Predictor	*t*	*p*
Castration—rubber rings (*N* = 103)	5.69 (0.13)	5.48 (0.10)	5.06 (0.10)		COM−LGE	*t*(860) = −2.75	0.006
					MIX−LGE	*t*(860) = −2.79	0.005
Castration—high tension bands (*N* = 90)	4.88 (0.27)	5.09 (0.20)	4.48 (0.20)		COM−LGE	*t*(330) = −0.35	0.729
					MIX−LGE	*t*(330) = −2.17	0.031
Supernumerary teat removal (*N* = 97)	4.90 (0.18)	4.02 (0.13)	3.35 (0.14)		COM−LGE	*t*(451) = −6.04	<0.001
					MIX−LGE	*t*(451) = −3.52	<0.001
Disbudding (*N* = 102)	4.83 (0.22)	4.32 (0.16)	4.22 (0.16)		COM−LGE	*t*(377) = −2.23	0.026
					MIX−LGE	*t*(377) = −0.44	0.659

Significance: *p* < 0.05; COM: companion animal practice, MIX: mixed animal practice, LGE: large animal practice; Direction of effect: negative *t* values indicate a reduction in perceived pain scores between the tested predictor (either COM or MIX) and LGE. ^a^ Perceived pain scores were measured on a 10-point scale (1 = no pain at all; 10 = most severe pain).

**Table 7:** The percentage and number of veterinarians that specified postprocedural pain durations across certain husbandry procedures.

Procedure	Postprocedural Pain Duration					
	None	<6 h	<12 h	<24 h	>24 h	>48 h
Castration— rubber rings (*N* = 104)	-	20.2% (*n* = 21)	7.7% (*n* = 8)	8.7% (*n* = 9)	27.9% (*n* = 29)	35.6% (*n* = 37)
Castration— high tension bands (*N* = 90)	-	17.8% (*n* = 16)	12.2% (*n* = 11)	6.7% (*n* = 6)	23.3% (*n* = 21)	40.0% (*n* = 36)
Supernumerary teat removal (*N* = 96)	2.1% (*n* = 2)	25.0% (*n* = 24)	20.8% (*n* = 20)	19.8% (*n* = 19)	15.6% (*n* = 15)	16.7% (*n* = 16)
Disbudding (*N* = 102)	-	5.9% (*n* = 6)	9.8% (*n* = 10)	9.8% (*n* = 10)	26.5% (*n* = 27)	48.0% (*n* = 49)

A dashed line represents a response option that was not selected by any of the respondents.

**Table 8:** A comparison of the impact of number of years since graduation ^a^ on the perceived postprocedural pain durations of veterinarians across certain husbandry procedures.

Procedure	*p*	OR (95% CI)
Castration—rubber rings (*N* = 98)	0.012	0.96 (0.93, 0.99)
Castration—high tension bands (*N* = 85)	0.045	0.96 (0.93, 1.00)
Supernumerary teat removal (*N* = 91)	0.036	0.96 (0.93, 1.00)
Disbudding (*N* = 96)	< 0.001	0.94 (0.90, 0.97)

Significance: *p* < 0.05; OR: odds ratio (per year since graduation); CI: confidence interval. ^a^ Number of years since graduation ranging between 0 and 58 (μ = 23.75, σ = 14.23).

**Table 9:** Mean (SD) perceived postprocedural pain durations of veterinarians across certain husbandry procedures by species emphasis.

	Species Emphasis					
Procedure	COM	MIX	LGE	Predictor	*p*	OR (95% CI)
Castration—rubber rings (*N* = 98)	5.18 (1.30)	4.46 (1.56)	4.20 (1.54)	COM–LGE	0.026	3.26 (1.15, 9.23)
				MIX–LGE	0.874	1.07 (0.47, 2.45)
Castration—high tension bands (*N* = 85)	5.16 (1.39)	4.64 (1.56)	4.09 (1.54)	COM–LGE	0.046	3.15 (1.02, 9.68)
				MIX–LGE	0.161	1.90 (0.77, 4.66)
Supernumerary teat removal (*N* = 91)	4.45 (1.32)	3.91 (1.38)	3.08 (1.40)	COM–LGE	0.002	5.23 (1.81, 15.07)
				MIX–LGE	0.008	3.29 (1.37, 7.89)
Disbudding (*N* = 96)	5.24 (0.94)	5.11 (1.24)	4.80 (1.29)	COM–LGE	0.843	1.11 (0.38, 3.25)
				MIX–LGE	0.629	1.24 (0.51, 3.00)

Significance: *p* < 0.05; SD: standard deviation; COM: companion animal practice, MIX: mixed animal practice, LGE: large animal practice; OR: odds ratio; CI: confidence interval.

**Table 10:** The percentage (number) of veterinarians that identified an age beyond which it is necessary to provide postoperative analgesic for calves across certain husbandry procedures.

Procedure	Any Age	>2 w	>8 w	>4 m	>6 m	None
Castration—rubber rings (*N* = 103)	55.3% (*n* = 57)	17.5% (*n* = 18)	12.6% (*n* = 13)	2.9% (*n* = 3)	1.0%(*n* = 1)	10.7% (*n* = 11)
Castration—high tension bands (*N* = 89)	64.0% (*n* = 57)	11.2% (*n* = 10)	16.9% (*n* = 15)	3.4% (*n* = 3)	0% (*n* = 0)	4.5% (*n* = 4)
Supernumerary teat removal (*N* = 97)	44.3% (*n* = 43)	17.5% (*n* = 17)	13.4% (*n* = 13)	10.3% (*n* = 10)	2.1% (*n* = 2)	12.4% (*n* = 12)
Disbudding (*N* = 101)	68.3% (*n* = 69)	6.9% (*n* = 7)	11.9% (*n* = 12)	5.0% (*n* = 5)	2.0% (*n* = 2)	5.9% (*n* = 6)

## Data Availability

The data presented in this study, along with access to the survey, are available on request from the corresponding author. The data are not publicly available due to privacy reasons.
